# Comprehensive management of hematopoietic stem cell transplantation complications: from infection prevention to immune microenvironment reconstruction

**DOI:** 10.3389/fimmu.2026.1740067

**Published:** 2026-03-11

**Authors:** Zhengrong Song, Xinzhi Han, Ziwei Zhou, Huan Hua, Fuxu Wang, Xuejun Zhang, Shupeng Wen

**Affiliations:** Department of Hematology, The Second Hospital of Hebei Medical University, Shijiazhuang, China

**Keywords:** graft-versus-host disease, hematopoietic stem cell transplantation, hepatic sinusoidal obstruction syndrome, immune reconstitution, infection prevention and control

## Abstract

This article systematically reviews the management of key complications in hematopoietic stem cell transplantation (HSCT), including infections, graft-versus-host disease (GVHD), and hepatic sinusoidal obstruction syndrome (VOD/SOS). It highlights the importance of optimizing conditioning regimens to reduce infection risk and discusses the role of novel antiviral agents like letermovir in transforming infection control. For GVHD, the pathogenesis involving effector and regulatory T-cell imbalances is analyzed, together with prevention strategies such as post-transplant cyclophosphamide with antithymocyte globulin and TCRαβ/CD19 depletion. Ruxolitinib is emphasized for steroid-refractory GVHD, and gut microbiota modulation is noted as a promising intervention. For VOD/SOS, early biomarker detection and defibrotide treatment are critical. The review also explores the impact of immune reconstitution on infection control, GVHD development, and relapse, and examines how emerging approaches, including single-cell sequencing, microbiome analysis, and artificial intelligence, can be applied in building whole-course risk management models. Future directions include developing intelligent platforms and personalized strategies to enhance long-term patient outcomes.

## 1 Introduction

Hematopoietic stem cell transplantation (HSCT) has served as a pivotal treatment for hematological disorders for over half a century. According to annual activity reports from the Center for International Blood and Marrow Transplant Research (CIBMTR), hematopoietic cell transplantation and cellular therapy continue to expand in clinical practice, with sustained growth in allogeneic transplantation and steadily improving survival outcomes over the past decade (1). However, the widespread application of allogeneic HSCT continues to be limited by transplant-related complications, which remain a major determinant of patient outcomes. Early post-transplant morbidity and mortality, particularly within the first 100 days, are primarily driven by severe infectious complications, acute graft-versus-host disease (GVHD), and hepatic sinusoidal obstruction syndrome (VOD/SOS). In the longer term, inadequate immune reconstitution and chronic transplant-related complications play an increasingly important role in shaping patient prognosis (2). Traditional HSCT complication management strategies suffer from fragmented and reactive limitations, focusing primarily on diagnosing and treating individual complications rather than systematically assessing and intervening in risks across the entire transplant cycle (3).

In recent years, advancements in molecular biology, immunology, and diagnostic technologies have led to breakthroughs in HSCT complication management. Mechanistic studies have deepened understanding of T-cell subset differentiation and regulation, offering new insights for GVHD management (4, 5); consensus diagnostic criteria and early detection strategies for sinusoidal obstruction syndrome/veno-occlusive disease (SOS/VOD) have enabled earlier recognition of this complication (6); and therapeutic innovations, including the clinical application of defibrotide, have significantly improved outcomes for patients with severe VOD/SOS after HSCT (7).

**Figure 1:**
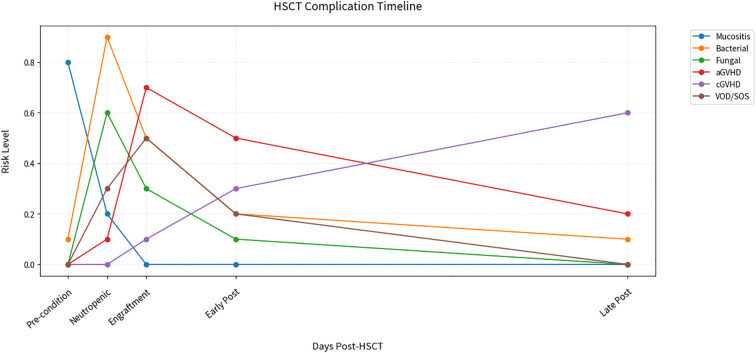
Timeline of comprehensive HSCT management. Line plots show dynamic risk levels for six complications across transplant phases. Key observations: Mucositis (blue) dominates pre-conditioning (peak risk 0.8). Infections bifurcate during neutropenia (bacterial 0.9 vs fungal 0.6). aGVHD (purple) peaks at engraftment (0.7) while cGVHD (orange) escalates post-Day 100. Data ranges reflect myeloablative regimens from EBMT 2023 (n>15,000). Phases defined per CIBMTR criteria.

Based on data from the 2023 European Society for Blood and Marrow Transplantation (EBMT) allogeneic HCT cohort, in which 56% of patients received myeloablative conditioning (n = 11,471 of 20,485) (8), we summarize a schematic timeline illustrating the typical temporal patterns of major HSCT-related complications across CIBMTR-defined transplant phases, including mucositis, infections, and graft-versus-host disease (GVHD) (**Figure 1**). This provides a critical basis for understanding complication epidemiology and formulating intervention strategies. Leveraging these temporal characteristics, modern management strategies are moving toward multi-dimensional integration, including stratified infection prevention, precise GVHD intervention, and early of vascular complications (9–11). Technological innovations—such as molecular monitoring, artificial intelligence, and microbiome analysis—play a key role in establishing a whole-course risk system. This review aims to construct an evidence-based framework for whole-course HSCT management by systematically analyzing the pathophysiological mechanisms of complications, immune reconstitution, and optimization of intervention strategies. This framework will provide phase-adapted monitoring protocols and personalized treatment decision trees for clinical practice, while identifying future research directions.

## 2 Clinical status and research progress in complication management

### 2.1 Infection prevention and control

HSCT recipients are highly susceptible to infections due to conditioning-induced myelosuppression, immunosuppressant use, and delayed or impaired immune reconstitution. Infections remain a leading cause of transplant-related mortality (12, 13), making effective infection prevention and control a cornerstone of whole-course HSCT management.

#### 2.1.1 Prevention and treatment of bacterial and fungal infections

HSCT patients are particularly vulnerable to bacterial and invasive fungal infections during the early post-transplant period, especially during neutropenia (14). Conditioning intensity is closely associated with infection risk after HSCT, as high-intensity regimens induce more profound myelosuppression and mucosal barrier injury, thereby increasing susceptibility to infections. Accordingly, reduced-intensity conditioning (RIC) has been increasingly adopted in selected populations and is generally associated with a lower burden of severe infectious complications. Beyond conditioning optimization, infection prevention after HSCT also relies on supportive strategies, including environmental control measures, antimicrobial prophylaxis, and biomarker- or imaging-guided early detection, which together facilitate timely intervention during the vulnerable post-transplant period. For example, a modified conditioning strategy incorporating pre-transplant immunosuppression has been reported to reduce transplant-related complications in transfusion-dependent β-thalassemia, indirectly highlighting the potential role of conditioning optimization in infection control (15).

For invasive fungal infections (IFIs), particularly invasive aspergillosis (IA), prophylaxis is of paramount importance due to their high mortality rate. Traditional strategies involve prophylactic therapy with broad-spectrum antifungal agents. The prophylactic efficacy of different antifungal drugs varies significantly, and their selection should be based on the stratification of patients’ risk for fungal infections. Clinical research data have shown the following: Fluconazole exhibits good activity against *Candida* species. Fluconazole has long been used as a primary agent for antifungal prophylaxis and remains recommended for patients at low risk of mold infection or in centers with a low incidence of such infections (16). However, it has no prophylactic effect on filamentous fungi such as *Aspergillus* and is suitable for low-risk patients. Voriconazole, a broad-spectrum triazole, covers both *Candida* and *Aspergillus* species. Data from the EBMT in 2019 showed that voriconazole prophylaxis reduced the incidence of IA from 15.6% to 6.2% in high-risk HSCT patients. However, attention should be paid to its visual toxicity and drug-drug interactions.

Recent network meta-analyses indicate that posaconazole substantially reduces the risk of invasive fungal infections compared with fluconazole-based prophylaxis in patients with hematological malignancies and HSCT recipients, with an estimated relative risk reduction of approximately 40–50%. A consistent benefit in preventing invasive aspergillosis has also been reported. Subgroup analyses further suggest that voriconazole may be particularly suitable for HSCT recipients, whereas posaconazole appears more beneficial in patients with acute myeloid leukemia or myelodysplastic syndrome (17, 18). However, its oral bioavailability is greatly affected by diet, and the intravenous formulation is more suitable for patients with impaired gastrointestinal function.

The treatment of IFIs requires individualized regimens based on the type of pathogen, site of infection, and immune status of the patient, with an emphasis on early targeted therapy: For *Candida* infections, fluconazole (400–800 mg daily) remains the first-line choice for infections caused by susceptible strains, with a clinical cure rate of 75%-80%. For fluconazole-resistant strains, caspofungin (70 mg on the first day, followed by 50 mg daily) or micafungin (100–150 mg daily) achieves a cure rate of 65%-70% (19). For *Aspergillus* infections, voriconazole (intravenous loading dose of 6 mg/kg every 12 hours on the first day, followed by 4 mg/kg every 12 hours) is the first-line treatment for IA. A global multicenter study showed that its clinical response rate was 53%, significantly higher than that of amphotericin B (31%) (20). Isavuconazole is an evidence-based alternative for the primary treatment of invasive mold disease, supported by the phase 3 SECURE trial demonstrating non-inferiority to voriconazole for all-cause mortality through day 42 (19% vs 20%), with fewer treatment-related adverse events (21). For rare fungi, liposomal amphotericin B (3–5 mg/kg daily) is the first choice, and combined surgical debridement can improve the cure rate. A retrospective study showed that for Mucor infections, the 12-week survival rate with liposomal amphotericin B combined with posaconazole maintenance therapy reached 52%, significantly higher than that with monotherapy (31%) (22).

In addition, adjunctive immunomodulatory therapies have been explored as supportive strategies for invasive fungal infections, including refractory invasive aspergillosis. These approaches primarily aim to enhance host antifungal immunity through immune stimulation, such as interferon-γ and colony-stimulating factors (e.g., G-CSF or GM-CSF). Current evidence suggests that such immunomodulatory interventions may be beneficial in selected immunocompromised patients; however, their clinical application remains limited by heterogeneous study designs and a lack of large, prospective trials (23). Current antifungal prophylaxis strategies in HSCT are largely guided by international consensus recommendations. In particular, the European Conference on Infections in Leukemia (ECIL) guidelines and the American Society for Transplantation and Cellular Therapy (ASTCT) consensus statements emphasize a risk-adapted approach, with mold-active agents recommended for high-risk patients and fluconazole-based strategies reserved for lower-risk settings (24, 25).

#### 2.1.2 Prevention and treatment of viral infections

Viral infections constitute another critical complication post-HSCT, with CMV, EBV, and respiratory viral infections being most prevalent. These not only cause direct disease but also indirectly increase transplant-related mortality by impairing immune reconstitution and triggering GVHD.

##### 2.1.2.1 Cytomegalovirus infection

CMV reactivation is one of the most common viral complications post-HSCT, strongly associated with increased non-relapse mortality (26). Traditional CMV management strategies include prophylaxis (routine antiviral use in high-risk patients early post-transplant) and preemptive therapy (treatment initiation upon detection of viremia before symptom onset). As noted, preemptive therapy effectively controls viral disease incidence, aligning with current clinical practice trends. Recent development of novel anti-CMV agents has expanded management options. Letermovir, a novel CMV terminase inhibitor approved for prophylaxis in high-risk CMV D+/R- kidney transplant recipients, has demonstrated efficacy in HSCT patients. Reviews by Razonable (27) and Grossi & Peghin (28) highlight letermovir’s efficacy and safety in CMV prevention, with lower myelosuppression and nephrotoxicity compared to traditional agents like ganciclovir and foscarnet. Maribavir, another novel agent, is used for refractory or drug-resistant CMV infections, showing superior efficacy and safety to traditional therapies in clinical trials (27). These agents significantly expand treatment options, particularly for patients intolerant of or resistant to traditional drugs.

In current clinical practice, CMV monitoring during HSCT primarily relies on regular quantitative polymerase chain reaction (qPCR)–based surveillance of CMV DNAemia, typically performed weekly during the early post-transplant period and extended in high-risk patients (28). Viral load kinetics are used to guide the initiation, escalation, or discontinuation of preemptive therapy, forming the cornerstone of CMV management in most transplant centers. Monitoring CMV-specific T-cell reconstitution is critical for predicting reactivation and guiding therapy. Jakharia et al. noted that CMV-specific T-cell-mediated immunity assays can identify patients with immune reconstitution and predict disease progression (12), though further prospective interventional studies are needed. Zhao et al. confirmed the role of CMV-specific T lymphocytes in predicting reactivation: low CMV-reactive CD4+ T-cell levels at 30 days post-transplant correlate with increased reactivation risk, while CD8+ T-cell levels at 60 days predict late reactivation (29). This supports personalized risk stratification and management via dynamic monitoring of these immune cell subsets. A proteomic screening study by Alexandersson et al. identified increased FCRL6 expression on γδ T cells in children post-allogeneic HSCT as associated with CMV reactivation, potentially offering new targets for immune-based interventions (30).

CMV infection can also cause organ-specific complications such as retinitis. Hiwarkar et al. found a high incidence of CMV retinitis in high-risk pediatric allogeneic HSCT recipients during immune reconstitution, recommending regular ophthalmic screening for this population (31). This underscores the systemic impact of CMV and the need for multidisciplinary management. Fan et al. further demonstrated that concurrent CMV infection and acute GVHD correlate with poor CD8+ T-cell reconstitution and adverse prognosis, indicating that CMV not only acts as an independent complication but also interacts with other conditions to exacerbate clinical burden (32).

##### 2.1.2.2 Epstein-Barr virus infection and post-transplant lymphoproliferative disorder

EBV infection is the primary driver of PTLD, a heterogeneous and potentially life-threatening complication after HSCT (33). The risk of EBV-PTLD is multifactorial and has been associated with profound T-cell immunosuppression, including T-cell–depleted grafts and the use of antithymocyte globulin (ATG) or alemtuzumab, as well as transplant-related factors such as unrelated or HLA-mismatched donors, graft-versus-host disease, reduced-intensity conditioning, cord blood transplantation, and second transplants. In addition, recipient- and donor-related factors, including older recipient age, donor EBV-seropositive/recipient-seronegative status, splenectomy, and infusion of mesenchymal stromal cells, have also been reported to increase PTLD risk (34).

Guidelines by Allen & Preiksaitis emphasize that pre-transplant EBV-seronegative recipients and primary EBV infection are key risk factors for EBV-associated PTLD (35). However, interpretation of EBV DNA monitoring in this setting remains challenging, owing to variability in intervention thresholds, differences in testing methodologies (e.g., whole blood versus peripheral blood mononuclear cell–based assays), and the lack of standardized protocols across centers. EBV-associated PTLD can be classified as proven or probable disease. Proven EBV-PTLD requires histopathological confirmation, including tissue biopsy with detection of EBV by Epstein–Barr virus–encoded RNA (EBER) *in situ* hybridization or viral antigen expression. When tissue biopsy is not feasible, a probable diagnosis may be established based on compatible clinical and radiological evidence of lymphoproliferation in conjunction with markedly elevated EBV DNA levels. Treatment strategies include reduction in immunosuppression (RIS), rituximab, and chemotherapy. Atallah-Yunes et al. noted that for PTLD following solid organ transplantation (SOT), rituximab with or without chemotherapy is often used sequentially after failure of RIS (36). In HSCT-associated PTLD, however, RIS alone or chemotherapy is typically less effective, and rituximab combined with RIS is considered the first-line standard therapy.

For patients with refractory or relapsed disease, second-line treatment options include adoptive cellular therapies, such as EBV-specific cytotoxic T lymphocytes (EBV-CTLs) or donor lymphocyte infusion (DLI), as well as chemotherapy with or without rituximab. More recently, off-the-shelf EBV-specific T-cell therapies, such as tabelecleucel (37), have emerged as promising options for rituximab-refractory EBV-PTLD, particularly in patients who are not candidates for donor-derived cellular therapies.

Novel strategies are being explored for refractory PTLD. EBV-specific cytotoxic T lymphocyte (CTL) infusions show promise (38, 39); Jing et al. reported successful treatment of refractory EBV-associated PTLD with Brentuximab Vedotin combined with allogeneic EBV-specific T lymphocytes (40). Additionally, immune checkpoint inhibitors like nivolumab have been trialed: Kassa et al. described complete remission in a child with primary central nervous system PTLD post-allogeneic stem cell transplantation after failure of all conventional therapies, using nivolumab monotherapy (41). These cases highlight the need for personalized PTLD treatment approaches.

##### 2.1.2.3 Respiratory viral infections

RVIs can cause severe complications and increased transplant-related mortality in HSCT patients. Trainor et al. found that pre-transplant respiratory viral PCR positivity correlates with increased 100-day transplant-related mortality in pediatric HSCT recipients (42). This underscores the importance of pre-transplant RVI screening to enable timely intervention or transplant plan adjustment, reducing risk.

In summary, infection prevention and control is a multi-dimensional, dynamically adjusted process encompassing pharmacologic prevention/treatment for known pathogens, precise monitoring and modulation of host immune status, and exploration of novel anti-infective agents and immunotherapies (43, 44). Future infection control will increasingly emphasize personalization and precision, integrating risk stratification, immune reconstitution status, and pathogen monitoring to optimize prevention and treatment.

### 2.2 Graft-versus-host disease

#### 2.2.1 Pathogenesis of GVHD

**Figure 2:**
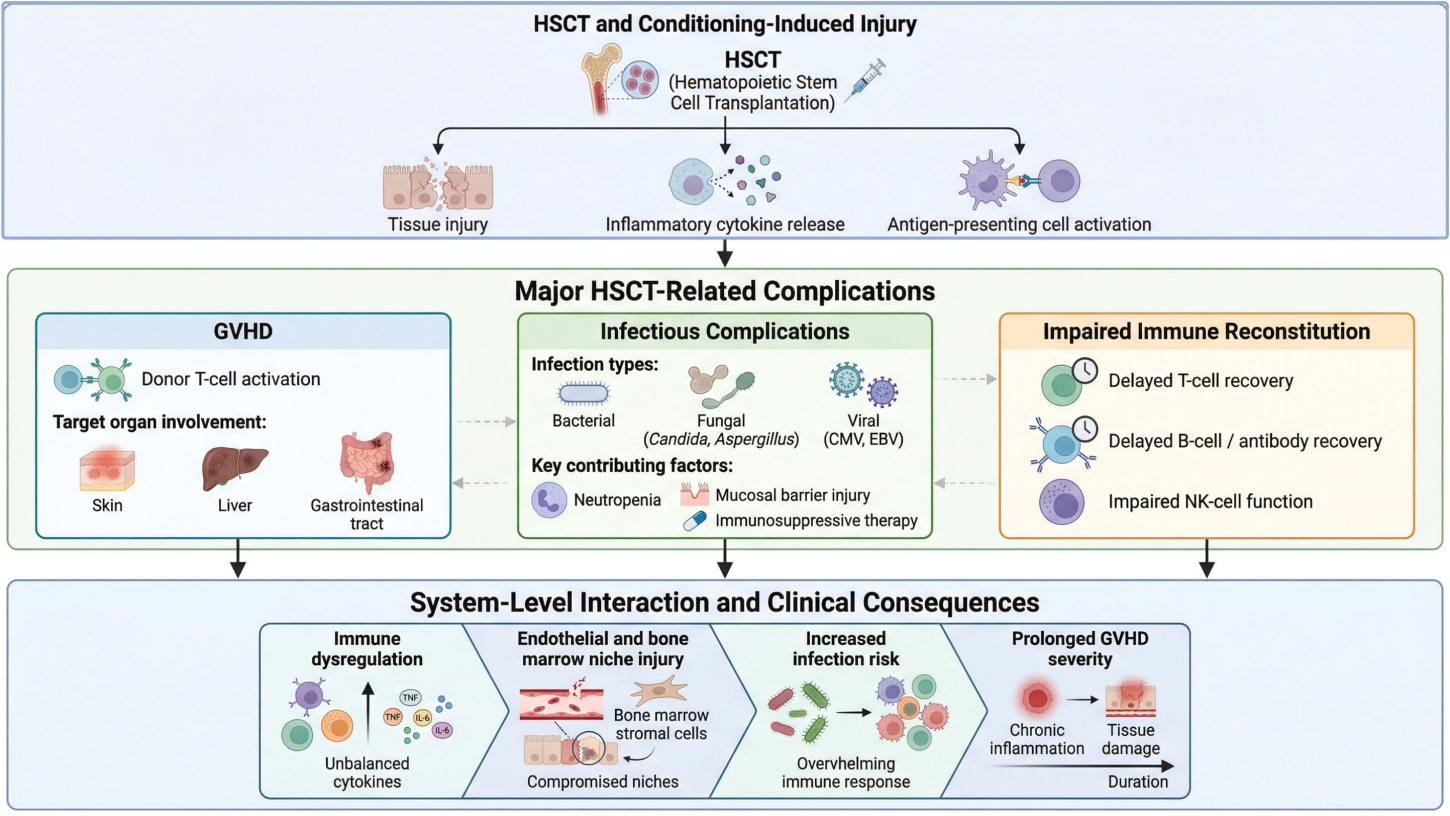
This schematic presents a systems-level overview of major complications following hematopoietic stem cell transplantation (HSCT). Conditioning-induced tissue injury initiates inflammatory cytokine release and antigen-presenting cell activation, driving the development of graft-versus-host disease (GVHD), infectious complications, and impaired immune reconstitution. These interrelated processes collectively contribute to immune dysregulation, endothelial and bone marrow niche injury, increased infection risk, and prolonged GVHD severity, highlighting the dynamic and interconnected nature of HSCT-related complications.

GVHD pathogenesis involves three key stages: conditioning-induced tissue damage releasing inflammatory mediators and activating antigen-presenting cells; donor T-cell recognition of alloantigens leading to activation and proliferation; and migration of activated T cells to target organs causing tissue damage (45, 46). Studies indicate that GVHD can affect not only traditionally recognized sites (skin, liver, gastrointestinal tract) but also the central nervous system, characterized by T-cell infiltration and neuronal injury (47) (**Figure 2**). Modern technologies have deepened understanding of GVHD mechanisms: single-cell sequencing reveals that effector T cells (Th1, Th17) promote GVHD, while Tregs exert protective effects (48); 29-color flow cytometry enables detailed analysis of NK and T-cell subsets (49). Recent research also links gut microbiota to GVHD, with specific microbial compositions potentially influencing disease development (50). These findings provide a basis for developing targeted therapies.

#### 2.2.2 GVHD prevention strategies

GVHD prevention is critical to the success of HSCT. Current prophylactic strategies encompass a broad range of approaches, including calcineurin inhibitor–based immunosuppression, *in vivo* or ex vivo T-cell depletion, and post-transplant cyclophosphamide (PTCy)–based platforms. Recent studies suggest that the addition of antithymocyte globulin (ATG) to PTCy-based regimens may further reduce the incidence of acute GVHD in selected transplant settings. For example, Koh et al. reported a lower rate of grade II–IV acute GVHD with PTCy plus ATG compared with PTCy alone, potentially reflecting the complementary effects of ATG on early T-cell activation and cytokine release. However, the use of ATG remains context-dependent and must be balanced against risks of delayed immune reconstitution and infection (51). In strategy selection, TCRαβ/CD19 depletion and PTCy regimens have distinct profiles: Lum et al. reported higher 3-year survival in the TCRαβ depletion group (78% vs 66%) in a multi-center study, while the PTCy group showed superior GVHD prevention (15% vs 6%) (52). Notably, ruxolitinib demonstrates unique preventive value in HSCT for myelofibrosis patients, with Wall et al. confirming its ability to optimize pre-transplant status and reduce GVHD risk (53). These studies inform personalized prevention strategy selection.

#### 2.2.3 Advances in GVHD diagnosis and treatment

GVHD diagnosis relies on clinical manifestations, histopathology, and biomarkers, with early diagnosis critical for timely intervention and improved prognosis. Corticosteroids remain first-line therapy for acute GVHD, with a response rate of approximately 50% (54). For steroid-refractory GVHD, the Janus kinase 2 (JAK2) inhibitor ruxolitinib has emerged as an effective second-line agent (55). Kujawska et al. emphasized that for patients refractory to both steroids and ruxolitinib—particularly those with severe gastrointestinal GVHD—validated treatment protocols are lacking, representing an unmet medical need (56). Regarding intestinal GVHD, Kujawska et al. highlighted the role of gut microbiota in GVHD pathophysiology, proposing it as a potential target for future therapies. Intestinal dysbiosis contributes significantly to GVHD development, and fecal microbiota transplantation may alleviate inflammatory responses by restoring microbial balance. Additionally, targeted therapies against specific cytokines or signaling pathways (IL-6 inhibitors) show promise: Xu et al. found that in haploidentical allo-HCT, the combination of rabbit ATG (10mg/kg) and low-dose PTCy (29mg/kg) not only reduced the incidence of severe aGVHD (from 18% to 5%, P = 0.003) but also significantly decreased serum IL-6 levels compared with ATG monotherapy, further supporting that IL-6 could serve as a surrogate marker for GVHD and a potential therapeutic target, consistent with the mechanism proposed in the original statement (57).

#### 2.2.4 Chronic GVHD

Chronic GVHD typically develops after day 100 post-transplant and can affect nearly all organ systems, representing a leading cause of morbidity and non-relapse mortality in long-term HSCT survivors (58). Its clinical manifestations range from mild skin involvement to severe organ dysfunction, significantly impacting quality of life. Vadakkel et al. (59) provided a detailed overview of multidisciplinary chronic GVHD management, emphasizing its wide-ranging effects on cardiovascular and metabolic health, bone density, endocrine function, sexual health, and ocular and pulmonary diseases. They noted that while glucocorticoids remain the mainstay of treatment, three oral agents have recently been approved for steroid-refractory chronic GVHD: ibrutinib, ruxolitinib, and belumosudil. These agents expand therapeutic options, particularly for patients unresponsive to steroids.

Chronic GVHD management must address not only disease control but also iatrogenic complications of immunosuppression (60). Long-term glucocorticoid use increases risks of osteoporosis, diabetes, and infections (61). Thus, multidisciplinary collaboration—including endocrinologists, dermatologists, ophthalmologists, pulmonologists, and gastroenterologists—is essential to develop comprehensive care plans that minimize disease burden and improve quality of life. Mascarenhas et al. also noted ruxolitinib’s efficacy in reducing acute and chronic GVHD and disease recurrence in myelofibrosis patients post-HSCT, further supporting its multifaceted role in GVHD management (62).

#### 2.2.5 GVHD and the immune microenvironment

GVHD development is closely linked to imbalances during immune reconstitution (63). While donor T-cell alloreactivity is central to GVHD pathogenesis, alterations in the recipient’s immune microenvironment—particularly endothelial and bone marrow niche damage—may exacerbate disease severity (64). Cao et al. found that acute GVHD patients exhibit reduced numbers and impaired function of bone marrow endothelial progenitor cells (EPCs), characterized by decreased migration and angiogenesis, elevated ROS levels, and increased apoptosis (65). They proposed that damaged bone marrow EPCs contribute to acute GVHD pathogenesis and associate with GVHD-mediated cytopenias, suggesting that improving bone marrow endothelial function could represent a novel therapeutic strategy. Furthermore, GVHD may impair immune reconstitution: Fan et al. observed poor CD8+ T-cell reconstitution and worse outcomes in patients with concurrent CMV infection and acute GVHD (32), indicating that GVHD can delay or compromise host immune responses to pathogens, increasing infection risk. Thus, GVHD management requires balancing immunosuppression and immune reconstitution to avoid excessive suppression while promoting effective pathogen-specific immunity (66). In summary, GVHD management is a complex, dynamic process requiring integration of prevention, early diagnosis, personalized treatment, and long-term complication care. Advances in mechanistic understanding and novel therapeutics are driving a shift toward precision medicine, leveraging multi-omics data to guide decisions and combining immunomodulatory strategies to control GVHD while preserving immune function and quality of life.

### 2.3 Hepatic sinusoidal obstruction syndrome

VOD/SOS is a severe, potentially fatal HSCT complication characterized by hepatomegaly, right upper quadrant pain, jaundice, and ascites (67). It arises when high-dose chemotherapy or radiation in conditioning regimens damages hepatic sinusoidal endothelial cells, disrupting sinusoidal wall integrity, causing obstruction, and leading to post sinusoidal portal hypertension.

#### 2.3.1 Pathogenesis and early diagnosis

The core pathophysiological mechanism of VOD/SOS involves hepatic sinusoidal endothelial cell injury and activation, inducing a procoagulant and antifibrinolytic state. This endothelial damage may extend beyond the liver, representing a systemic endothelial injury syndrome. Al Jefri et al. comprehensively reviewed VOD/SOS pathogenesis, clinical manifestations, diagnostic criteria, risk factors, prevention, and treatment, noting recent updates to diagnostic tools for both pediatric and adult populations (68). Early diagnosis is critical for VOD/SOS prognosis, as severe disease (often with multi-organ dysfunction) carries mortality exceeding 80% (69). Serum biomarkers such as plasma plasminogen activator inhibitor-1 (PAI-1), von Willebrand factor (vWF), and serum bilirubin have been widely studied as early predictors. Dynamic monitoring of these biomarkers combined with imaging enables identification of high-risk patients before symptom onset, facilitating timely intervention.

##### 2.3.1.1 Clinical manifestations and diagnostic criteria of VOD/SOS

Clinically, VOD/SOS typically presents with unexplained weight gain not attributable to fluid overload, peripheral edema or ascites, painful hepatomegaly, and jaundice. Importantly, anicteric VOD/SOS is increasingly recognized in both pediatric and adult patients, indicating that the absence of hyperbilirubinemia does not exclude the diagnosis, particularly in early or late-onset disease (70).

Several diagnostic criteria have been proposed to standardize VOD/SOS diagnosis. The classic Seattle and Baltimore criteria are primarily based on clinical features and bilirubin elevation within defined post-transplant time windows. More recently, EBMT criteria have expanded this framework by incorporating late-onset and anicteric presentations, as well as severity grading, thereby facilitating earlier recognition and risk stratification (71).

Imaging serves as an important adjunct to clinical assessment. Doppler ultrasound can identify hepatomegaly, ascites, and alterations in portal venous flow, while hepatic elastography provides a noninvasive assessment of liver stiffness associated with sinusoidal congestion. In selected cases, hemodynamic evaluation, including measurement of the hepatic venous pressure gradient, may further support severity assessment (72).

Beyond pharmacologic interventions, non-pharmacologic preventive strategies are increasingly emphasized, including optimization of iron overload through chelation prior to transplantation, evaluation and management of viral hepatitis, careful selection of conditioning regimens (e.g., treosulfan over busulfan in high-risk patients), and awareness of SOS-associated risks related to agents such as gemtuzumab ozogamicin and sirolimus (73).

#### 2.3.2 Treatment and prevention

##### 2.3.2.1 Defibrotide

Defibrotide is currently the only approved agent for treating severe VOD/SOS post-HSCT. Its complex mechanism of action includes endothelial protection, restoration of fibrinolytic activity, and anti-inflammatory effects. Richardson et al. demonstrated that earlier defibrotide initiation after VOD/SOS diagnosis significantly improves 100-day post-transplant survival: among 573 patients analyzed, ~30% started treatment on the day of diagnosis, and over 90% within 7 days, with earlier initiation associated with better outcomes (74). This finding strongly supports prompt defibrotide administration upon VOD/SOS diagnosis.

##### 2.3.2.2 Preventive strategies

Given the severity of VOD/SOS—particularly with multi-organ involvement—preventive strategies remain under investigation (71). Risk factor identification is foundational, including pre-existing liver disease, second transplants, specific conditioning regimens, GVHD, and genetic predispositions. Prophylactic measures for high-risk patients may include conditioning intensity adjustment, ursodeoxycholic acid, or low-dose heparin, though their efficacy and safety require further high-quality clinical studies.

Faraci et al. (75) attributed reduced VOD/SOS-related mortality in recent years partly to improved intensive care and multidisciplinary approaches, emphasizing that management relies not only on specific pharmacotherapy but also on close collaboration among hepatologists, intensivists, hematologists, and radiologists to implement comprehensive care plans.

##### 2.3.2.3 Endothelial cell injury and repair

VOD/SOS pathogenesis shares endothelial cell injury mechanisms with other post-transplant complications, such as thrombotic microangiopathy (TMA) and poor graft function (PGF). Meri et al. explored the role of the complement system in HSCT-TMA, noting complement activation post-HSCT interacts with coagulation and inflammatory pathways to induce endothelial damage (76). While focused on TMA, this mechanistic insight enhances understanding of VOD/SOS.

Kong et al. and Wang et al. linked bone marrow endothelial cell (BM EC) damage to PGF and prolonged thrombocytopenia (PT), finding that pre-transplant BM EC depletion increases PGF and PT risk. Prophylactic oral N-acetylcysteine (NAC) reduced these risks by improving BM EC reconstruction and reducing ROS levels (77, 78). Though focused on hematopoietic function, these studies highlight the universal role of endothelial injury in transplant complications, suggesting endothelial protection strategies may benefit multiple conditions, including VOD/SOS.

Thus, VOD/SOS management is comprehensive, encompassing risk assessment, early, timely diagnosis, and specific treatment. Future research should focus on developing more sensitive early diagnostic biomarkers, exploring novel endothelial protectants, and optimizing defibrotide use to further reduce VOD/SOS incidence and mortality.

## 3 Immune microenvironment reconstitution and construction of whole-course management models

HSCT success depends not only on engraftment and disease control but also on post-transplant reconstitution and homeostasis of the host immune system. Immune reconstitution quality directly influences infection resistance, GVHD development, and tumor recurrence risk. Concurrently, advances in high-throughput technologies and artificial intelligence have driven integration of multi-dimensional data to build whole-course management models—an important trend in HSCT.

### 3.1 Dynamic monitoring and intervention of immune reconstitution

Post-transplant immune reconstitution is a complex, prolonged process involving quantitative and functional recovery of T cells, B cells, NK cells, and myeloid cells. Recovery rates and patterns vary among cell subsets, influenced by conditioning regimens, graft type, GVHD prophylaxis, and complications.

#### 3.1.1 T-cell immune reconstitution

T cells are central to post-HSCT immune reconstitution, with their quantity and function critical for anti-infective and anti-tumor immunity (79, 80). Fuji et al. reviewed pathogen-specific T-cell monitoring methods, noting their potential to identify high-risk patients and guide immunotherapy (81). Lyu et al. confirmed the role of CMV-specific CD4+ and CD8+ T cells in predicting CMV reactivation, emphasizing dynamic monitoring for personalized risk stratification (82). Link et al. further linked concurrent CMV infection and acute GVHD to delayed CD8+ T-cell reconstitution and poor prognosis, highlighting GVHD and infection as negative regulators of T-cell recovery (9). Stuehler et al. observed reduced CD4+ and CD8+ T-cell counts, antigen-specific T-cell responses, and PMN killing capacity in IA patients within 12 months post-transplant, indicating T-cell dysfunction as a key driver of post-HSCT infection susceptibility. Accelerating early, effective T-cell reconstitution is therefore critical for reducing infection risk (13).

#### 3.1.2 NK cell and myeloid cell reconstitution

Beyond T cells, NK cells and myeloid cells are vital for post-HSCT immune function. Fernández et al. found persistently low NK cell counts in IA patients, with lower counts associated with poor outcomes—underscoring NK cells’ role in antifungal immunity. Myeloid cells, particularly neutrophils, form the first line of defense against bacterial and fungal infections (83).

#### 3.1.3 Interventions to enhance immune reconstitution

Multiple strategies are being explored to accelerate immune reconstitution. Dal Collo et al. developed a CD117-targeting bispecific T-cell engager (BTCE) and demonstrated, in humanized mouse models, its ability to achieve rapid bone marrow conditioning and leukemia clearance while enabling efficient HSCT (84). This approach aims to reduce traditional conditioning toxicity, potentially facilitating faster immune reconstitution. Additionally, Kong et al. and Wang et al. found NAC promotes hematopoietic reconstitution by improving bone marrow endothelial function, indirectly supporting strategies to optimize the bone marrow niche for immune cell production (77, 78).

## 4 Application of emerging technologies in whole-course management

With the increasing complexity of hematopoietic stem cell transplantation, a range of emerging technologies has been incorporated into clinical research and practice to improve the understanding, prediction, and management of transplant-related complications. These approaches span multiple levels, including cellular and molecular profiling, host–microbiome interactions, computational modeling, and precision pharmacology, and collectively contribute to more refined whole-course management strategies. The following sections summarize representative technological advances and their potential roles in improving risk stratification, mechanistic insight, and clinical decision-making after HSCT.

### 4.1 Single-cell sequencing

Single-cell sequencing enables analysis of cellular heterogeneity at single-cell resolution, offering unprecedented insights into complex post-HSCT immune reconstitution and complication mechanisms (85, 86). Single-cell RNA sequencing can identify abnormally activated T-cell subsets or clonal expansions during GVHD, guiding precise targeted therapy to avoid over-suppression of normal immune cells. While not single-cell sequencing, Alexandersson et al. proteomic study identified increased FCRL6 expression on γδ T cells associated with CMV reactivation, exemplifying high-throughput technologies’ potential to discover novel biomarkers and therapeutic targets (30).

### 4.2 Microbiome analysis

Gut microbiota play critical roles in post-HSCT immune reconstitution, GVHD development, and infection risk (87). Beyond intestinal GVHD, increasing evidence suggests that microbial diversity and compositional balance are also closely associated with systemic immune recovery, susceptibility to bloodstream infections, and overall transplant outcomes, highlighting the broader clinical relevance of addressing dysbiosis in HSCT recipients (88).

Microbiome analysis technologies enable comprehensive assessment of gut microbial composition and function, providing a basis for modulating the microbiome via fecal microbiota transplantation (FMT) or other approaches (89). Such strategies aim to restore microbial diversity, strengthen epithelial barrier integrity, and rebalance immune signaling, thereby potentially mitigating inflammatory and infectious complications after transplantation.

In intestinal GVHD, restoring microbial diversity and balance may alleviate inflammation and reduce infection risk (90). Future research will further explore associations between specific microbiota and HSCT outcomes, developing more precise microbiome-based interventions. However, current evidence is largely observational, and challenges related to causal inference, inter-individual variability, and the safety and standardization of microbiota-targeted interventions remain to be addressed.

### 4.3 Artificial intelligence prediction

AI and machine learning (ML) are increasingly applied in HSCT to predict complications, enable early diagnosis, and support treatment decisions (91, 92). Garuffo et al. noted ML’s potential to improve HSCT decision-making—including donor selection, conditioning regimens, and outcome prediction. Techniques such as decision trees, random forests, and neural networks can optimize donor matching, predict mortality and recurrence, and stratify GVHD risk (93). Integrating genomic and biomarker data with ML will enable personalized treatment strategies.

### 4.4 Therapeutic drug monitoring

In addition to the aforementioned emerging technologies, traditional therapeutic drug monitoring (TDM) also plays a crucial role in the whole-course management of HSCT (94). Vanderstoep et al. reviewed TDM of conditioning agents in pediatric allogeneic HSCT, noting that TDM is valuable for optimizing drug dosages, reducing toxicity while maintaining efficacy—particularly in pediatric patients with greater pharmacokinetic variability (95). Kaye et al. also emphasized the key role of TDM and pharmacogenomics for immunosuppressants such as tacrolimus and mycophenolate mofetil in mitigating drug toxicity (96). By maintaining drug concentrations within the therapeutic window, TDM minimizes adverse effects like nephrotoxicity and neurotoxicity while ensuring adequate immunosuppressive efficacy. This underscores the importance of precision pharmacology in the whole-course management of HSCT.

## 5 Conclusion

HSCT as a pivotal curative therapy for various hematological disorders, is inextricably linked to the effective management of complications for improving its efficacy. This review comprehensively summarizes recent advances in major post-HSCT complications, including infections, GVHD, VOD/SOS and post-PTLD, while exploring the critical role of immune microenvironment reconstitution and the application of emerging technologies in whole-course management models.

In infection prevention and control, studies have confirmed a close correlation between conditioning intensity and infection risk. Stratified anti-infective strategies, particularly enhanced prophylaxis for high-risk patients, have effectively reduced the incidence of invasive fungal infections (97, 98). For CMV reactivation, the combination of preemptive therapy and novel antiviral agents has significantly improved viral disease control. Meanwhile, dynamic monitoring of CMV-specific T-cell reconstitution provides a crucial basis for personalized risk assessment and treatment. For EBV-associated PTLD, strategies such as immunosuppressant reduction, rituximab, and EBV-specific cytotoxic T-cell infusions offer new hope for patients (99, 100). The importance of pre-transplant respiratory virus screening has also become increasingly evident, contributing to reduced transplant-related mortality.

Significant breakthroughs have been achieved in GVHD management. In acute GVHD, single-cell sequencing has deeply revealed the key role of specific T-cell subsets in disease pathogenesis, laying the foundation for developing personalized treatment regimens and significantly improving survival rates in steroid-refractory patients (101). The dual T-cell suppression strategy of PTCy combined with ATG has demonstrated excellent GVHD prevention efficacy in haploidentical transplantation while reducing infection risk (102, 103). For chronic GVHD, multidisciplinary management combined with novel oral agents has effectively alleviated disease burden. Additionally, the promising potential of fecal microbiota transplantation in treating intestinal GVHD provides new insights into immunomodulation (104). Notable progress has been made in the early diagnosis of VOD/SOS. A detection protocol combining ultrasound elastography and serum biomarkers enables pre-symptomatic early warning (105). Prospective studies have confirmed that initiating defibrotide therapy within a specific time window significantly improves prognosis, emphasizing the importance of timely intervention.

This review innovatively constructs a whole-course management model integrating single-cell sequencing, microbiome analysis, and artificial intelligence prediction. By combining multi-omics data with clinical information, it achieves precise prediction, early warning, and personalized treatment of complications. Despite significant advances in HSCT complication management, numerous challenges and research gaps remain. Firstly, most existing studies focus on single complications; future research should explore interactions and shared pathogenesis between different complications to develop more comprehensive intervention strategies. Secondly, although new drugs continue to emerge, some patients remain resistant or intolerant to current therapies, necessitating the development of more targeted, less toxic agents. Thirdly, the discovery and validation of biomarkers require further enhancement to enable more precise risk stratification and earlier diagnosis of HSCT-related complications. For example, although ultrasound elastography and serum biomarkers have shown potential value in the early warning of VOD/SOS, their universality and diagnostic accuracy in large-scale clinical practice remain insufficiently established, owing to limited external validation, heterogeneous cutoff definitions, and variability across transplant platforms and patient populations (106). Consequently, robust multicenter prospective studies are needed to standardize biomarker panels and to clarify their predictive performance across different conditioning regimens and risk groups. Fourthly, the complexity of immune reconstitution after HSCT demands more sophisticated monitoring tools and effective immunomodulatory strategies, as immune recovery is highly dynamic and influenced by graft source, conditioning intensity, and post-transplant immunosuppression. Current monitoring approaches, which largely rely on static immune cell counts, often fail to capture functional immune competence or immune balance. Integrating longitudinal immune profiling, functional assays, and high-dimensional technologies may therefore be necessary to better guide interventions that accelerate immune recovery while minimizing the risk of alloreactivity. Finally, managing long-term complications and improving patients’ quality of life remain critical challenges, underscoring the need for more robust long-term follow-up systems, multidisciplinary care models, and outcome measures that extend beyond survival to encompass functional recovery and overall well-being.

Building on the above evidence, future directions in HSCT complication management should focus on actionable strategies for clinical implementation and clearly defined research priorities, including the following areas: (1) Deep integration of multi-omics data and artificial intelligence-driven precision medicine: Combining genomics, transcriptomics, proteomics, metabolomics, and microbiomics with artificial intelligence and machine learning algorithms to build more powerful predictive models and decision-support systems, enabling truly personalized risk assessment and treatment planning. (2) Development of novel targeted therapies and cell-based therapies: Focusing on core pathophysiological mechanisms of complications to develop more specific, less toxic targeted drugs and cell therapy products, such as precision immunomodulators, endothelial protectants, and gene-editing-based cell therapies. (3) Refined regulation of the immune microenvironment: Investigating the roles of bone marrow and intestinal microenvironments in immune reconstitution and complication pathogenesis, exploring strategies to promote hematopoietic recovery, suppress GVHD, and enhance anti-infective capacity through microenvironment intervention. (4) Optimization and advancement of preventive strategies: Developing more effective preventive interventions based on high-risk factors and early biomarkers, shifting complication management to pre-transplant and early post-transplant phases to minimize incidence and severity. (5) Long-term follow-up and quality of life improvement: Establishing comprehensive long-term follow-up systems to monitor long-term complications and quality of life, providing holistic rehabilitation, psychological, and social support to ensure optimal long-term survival and quality of life for post-transplant patients. Through continuous scientific research and innovation in clinical practice, we are confident that the whole-course management of HSCT complications will continue to improve, bringing curative hope and high-quality life to more patients.
